# Empagliflozin improved systolic blood pressure, endothelial dysfunction and heart remodeling in the metabolic syndrome ZSF1 rat

**DOI:** 10.1186/s12933-020-00997-7

**Published:** 2020-02-18

**Authors:** Sin-Hee Park, Muhammad Akmal Farooq, Sébastien Gaertner, Christophe Bruckert, Abdul Wahid Qureshi, Hyun-Ho Lee, Djamel Benrahla, Brigitte Pollet, Dominique Stephan, Patrick Ohlmann, Jean-Marc Lessinger, Eric Mayoux, Cyril Auger, Olivier Morel, Valérie B. Schini-Kerth

**Affiliations:** 1 INSERM (French National Institute of Health and Medical Research), UMR 1260, Regenerative Nanomedicine, FMTS, Strasbourg, France; 2 Hôpitaux Universitaires de Strasbourg, Service des Maladies Vasculaires - Hypertension Artérielle, Strasbourg, France; 3 UMR CNRS 7021 Laboratoire de Bioimagerie et Pathologies, Strasbourg, France; 4 Hôpitaux Universitaires de Strasbourg, Service de Cardiologie, Strasbourg, France; 5 Laboratory of Biochemistry and Molecular Biology, Hôpitaux Universitaires de Strasbourg, Strasbourg, France; 6 Boehringer Ingelheim Pharma GmbH & Co. KG, Biberach, Germany

**Keywords:** Empagliflozin, SGLT2, Endothelial function, Heart function, Heart structure, Senescence, ZSF1, Metabolic syndrome

## Abstract

**Background:**

Empagliflozin (empa), a selective sodium–glucose cotransporter (SGLT)2 inhibitor, reduced cardiovascular mortality and hospitalization for heart failure in patients with type 2 diabetes at high cardiovascular risk independent of glycemic control. The cardiovascular protective effect of empa was evaluated in an experimental model of metabolic syndrome, the obese ZSF1 rat, and its’ lean control.

**Methods:**

Lean and obese ZSF1 rats were either non-treated or treated with empa (30 mg/kg/day) for 6 weeks. Vascular reactivity was assessed using mesenteric artery rings, systolic blood pressure by tail-cuff sphygmomanometry, heart function and structural changes by echocardiography, and protein expression levels by Western blot analysis.

**Results:**

Empa treatment reduced blood glucose levels from 275 to 196 mg/dl in obese ZSF1 rats whereas normoglycemia (134 mg/dl) was present in control lean ZSF1 rats and was unaffected by empa. Obese ZSF1 rats showed increased systolic blood pressure, and blunted endothelium-dependent relaxations associated with the appearance of endothelium-dependent contractile responses (EDCFs) compared to control lean rats. These effects were prevented by the empa treatment. Obese ZSF1 rats showed increased weight of the heart and of the left ventricle volume without the presence of diastolic or systolic dysfunction, which were improved by the empa treatment. An increased expression level of senescence markers (p53, p21, p16), tissue factor, VCAM-1, SGLT1 and SGLT2 and a down-regulation of eNOS were observed in the aortic inner curvature compared to the outer one in the control lean rats, which were prevented by the empa treatment. In the obese ZSF1 rats, no such effects were observed. The empa treatment reduced the increased body weight and weight of lungs, spleen, liver and perirenal fat, hyperglycemia and the increased levels of total cholesterol and triglycerides in obese ZSF1 rats, and increased blood ketone levels and urinary glucose excretion in control lean and obese ZSF1 rats.

**Conclusion:**

Empa reduced glucose levels by 28% and improved both endothelial function and cardiac remodeling in the obese ZSF1 rat. Empa also reduced the increased expression level of senescence, and atherothrombotic markers at arterial sites at risk in the control lean, but not obese, ZSF1 rat.

## Background

Metabolic syndrome is a critical health state comprising central obesity, dyslipidemia, hypertension, and glucose intolerance that carries increased risk for both type 2 diabetes (T2D) and cardiovascular disease (CVD) [1–4]. Over the past three decades, the prevalence rate of metabolic syndrome has increased rapidly with age worldwide and its prevalence ranges from < 10% up to 84% [5, 6]. The direct and indirect effects of hyperglycemia in T2D on the vascular tree promote both macrovascular complications including stroke, coronary artery diseases, and peripheral vascular diseases, and microvascular complications including diabetic retinopathy, nephropathy, and neuropathy [7]. One of the earliest impact of hyperglycemia on the cardiovascular system leads to the induction of an endothelial dysfunction characterized by a reduced nitric oxide (NO) component and often also endothelium-dependent hyperpolarization (EDH) component, and the induction of endothelium-dependent contractile responses (EDCFs) [8–14]. Even though the entire vascular tree is subjected to cardiovascular risk factors, phenotypic alterations leading to a dysfunctional endothelium are revealed initially at specific regions of arteries such as curvatures and bifurcations, where disturbed flow and low shear stress take place and which are prone to early atherogenesis. Senescent endothelial cells (ECs), which are characterized by endothelial nitric oxide synthase (eNOS) down-regulation and a reduced formation of NO, are observed in the human aortic arch [15] and coronary artery at sites overlying atherosclerotic plaques [16]. The fact that the selective expression of the senescence marker p53 in the endothelium leads to diminished endothelium-dependent relaxations and NO formation in aortic rings of rats [17] suggests that the induction of cellular senescence acts as a critical upstream signaling pathway to promote endothelial dysfunction.

Gliflozins including empagliflozin, dapagliflozin, and canagliflozin are a novel class of antidiabetic agents used for the treatment of T2D that selectively inhibit the sodium–glucose cotransporter (SGLT)2 to prevent glucose reabsorption in the renal proximal tubule. In the cardiovascular EMPA-REG OUTCOME trial including 7020 T2D patients with established CVD, empagliflozin reduced cardiovascular death by 38% and heart failure (HF) hospitalization by 35% [18]. In T2D patients with established CVD, canagliflozin reduced the composite of cardiovascular cause of death including nonfatal myocardial infarction or nonfatal stroke by 14% and HF hospitalization by 33% [19], and dapagliflozin lowered the rate of cardiovascular death or HF hospitalization, with a 27% lower rate of HF hospitalization [20]. Subsequent analysis of the EMPA-REG OUTCOME data indicated that the cardioprotective effect of empagliflozin appears to be independent of glycemic control [21], suggesting that mechanisms, in addition to glycemic control, are involved. Such a concept is also supported by experimental studies indicating that empagliflozin improved endothelium-dependent relaxations in streptozotocin-induced diabetic rats [22], and hemodynamics in a hypertensive heart failure rat model [23], decreased left ventricular weight, cardiomyocytes size, and cardiac interstitial fibrosis and macrophage infiltration in genetic prediabetic rats with metabolic syndrome [24]. Empagliflozin also significantly reduced left ventricle (LV) mass and LV systolic dilatation [25], decreased glucotoxicity thereby preventing the development of endothelial dysfunction, and reduced oxidative stress, cardiac fibrosis and exhibited anti-inflammatory effects in ZDF rats and mice [26, 27]. Moreover, empagliflozin reduced the levels of CD36 and cardiotoxic lipids improving autophagy in the hearts of ZDF rats [28], and decreased aortic stiffness, renal resistivity index and kidney injury in T2D female mice [29]. Finally, empagliflozin ameliorated kidney injury in T2D female mice by promoting glycosuria, and possibly by reducing systemic and renal artery stiffness [29]. In addition, a redox-sensitive up-regulation of SGLT1 and 2 has been observed in coronary artery ECs in response to high glucose and H_2_O_2_ leading to enhanced glucose uptake and induction of atherothrombotic responses [30]. Altogether, these findings support the concept that gliflozins protect the cardiovascular system, besides the improvement of the glycemic control, possibly also by targeting the cardiovascular and, in particular, the pivotal protective endothelial function.

Therefore, the aim of the present study was to investigate the effect of a 6-week oral intake of empagliflozin on the cardiovascular system in the obese ZSF1 rat, an established experimental model of metabolic syndrome with preserved ejection fraction (HFpEF) and systolic hypertension, and its’ lean control. In particular, the effect of the empagliflozin treatment was evaluated on: (1) the metabolic status, (2) the level of systolic blood pressure, (3) the endothelial function in the isolated mesenteric artery, and (4) the structure and function of the heart.

## Materials and methods

### Materials

Empagliflozin was provided by Boehringer Ingelheim Pharma GmbH & Co KG (Biberach an der Riss, Germany). All other chemicals were from Sigma-Aldrich (Sigma-Aldrich Chemie SARL, St Quentin Fallavier, France) unless otherwise specified.

### Animals and in vivo treatment

The experimental model of metabolic syndrome studied was the obese Zucker diabetic fatty/Spontaneously hypertensive heart failure F1 hybrid (ZSF1)-HFpEF rats and its’ lean control. A total number of 20 obese ZSF1 rats and respective 20 lean control rats were obtained from Charles River Laboratories. At an age of 12 weeks, rats were divided into four groups of n = 10 per group: lean control rats, lean control rats + empagliflozin (30 mg/kg/day), obese ZSF1 rats and obese ZSF1 rats + empagliflozin (30 mg/kg/day) provided in the feed. After a 6-week treatment period, rats were euthanized by IP injection of an overdose of ketamine and xylazine (120 and 20 mg/kg, i.p.).

### Biochemical analysis

Urine samples were collected from rats for 24 h in metabolic cages on the day before sacrifice and blood glucose and ketone levels were assessed in tail bleed from overnight (15 h) fasted rats using the blood glucose and β-ketone meter (GLUCOFIX^®^ Premium, A. Menarini diagnostics) shortly after being euthanized. Blood samples were collected by terminal cardiac puncture in heparin containing tubes and plasma was prepared after centrifugation at 7000 rpm for 10 min at room temperature. Thereafter aliquots were stored at − 80 °C until use. Plasma parameters were determined using an Advia 20400 automatic analyzer (Siemens Healthineers) and plasma lipid levels using an AU480 chemistry analyzer (Beckman Coulter). Total cholesterol was assessed using OSR6116 (Enzymatic Color Test CHOD-PAP Method), HDL using cholesterol OSR6187 (Immunoinhibition/enzymatic color test), LDL cholesterol using OSR6183 (enzymatic color test CHOD-PAP method) and triglycerides using OSR61118 (enzymatic color test GPO-PAP method).

### Blood pressure measurements

Systolic blood pressure was determined by tail-cuff sphygmomanometry twice weekly during 5 weeks using the blood pressure analysis system (BP-2000 Serie II, Visitech Systems). Prior to start of blood pressure monitoring, rats were trained daily for 1 week to get used to the system.

### Echocardiography

After 5 weeks of treatment, rats were anaesthetized by inhalation of isoflurane (5% induction and 2% for maintenance, 5 l/min of air plus 2 l/min of O_2_). Cardiac structure and function were determined by transthoracic echocardiography using the Phillips Sonos 5500 machine equipped with a probe 12 MHz transducer. Two-dimensional short axis views of the left ventricle and M-mode tracings were recorded through anterior and posterior left ventricular walls at the papillary muscle level.

Morphological characterization of the cardiac left ventricle was performed following the determination of the parameters: left ventricular end-diastolic diameter, left ventricular end-systolic diameter, and posterior diastolic wall thickness (PWT). Left ventricular volume, E/E′ ratio, cardiac output and left ventricular ejection fraction were subsequently obtained from these parameters.

### Western blot analysis

Inner and outer segments of the aortic arch were homogenized in extraction buffer [composition in mM: Tris/HCl 20 (pH 7.5), NaCl 150, Na_3_VO_4_ 1, Na_4_P_2_O_7_ 10, NaF 20, okadaic acid 0.01, 1% Triton X-100 and protease inhibitor cocktail (Complete Mini, Roche)]. Total proteins (15 μg) were separated on 8 or 12% SDS polyacrylamide gels and transferred electrophoretically onto nitrocellulose membrane (GE Healthcare Life Sciences). After blocking with 5% bovine serum albumin in Tris-buffered saline (TBS) containing 0.1% Tween 20 for 1 h at room temperature, membranes were incubated with primary antibodies against rabbit polyclonal anti-p53 (1:1000; Santa Cruz Biotechnology; sc-6243), mouse monoclonal anti-p21 (1:1000; Santa Cruz Biotechnology; sc-817), rabbit polyclonal anti-p16-INK4 (1:500; Abbiotec; 250804), murine monoclonal anti-tissue factor (TF; 1:1000; Sekisui Diagnostics; 4509), mouse monoclonal anti-eNOS/NOS (1:5000; BD Transduction Laboratories; 610297), rabbit monoclonal anti-VCAM-1 (1:10,000; Abcam; ab134047), rabbit polyclonal anti-SGLT1 (1:1000; Santa Cruz Biotechnology; sc-98974), rabbit polyclonal anti-SGLT2 (1:1000; Santa Cruz Biotechnology; sc-98975) or mouse monoclonal anti-β-tubulin (1:20,000; Sigma-Aldrich; T7816) overnight at 4 °C. After washing, membranes were incubated with the secondary antibody (peroxidase-labeled anti-rabbit or anti-mouse immunoglobulin G; 1:10,000; Cell Signaling Technology; #7074, #7076, respectively) for 1 h at room temperature. The immunoreactive bands were developed by enhanced chemiluminescence (ECL, Amersham) using ImageQuant LAS 4000 (GE Healthcare).

### Vascular reactivity study

Vascular reactivity study was performed using the main mesenteric artery as described previously [31]. Briefly, the main superior mesenteric arteries were cleaned of connective tissue, and cut into rings (2–3 mm in length). Then, rings were suspended in organ baths containing oxygenated (95% O_2_, 5% CO_2_) Krebs bicarbonate solution (mM: NaCl 119, KCl 4.7, KH_2_PO_4_ 1.18, MgSO_4_ 1.18, CaCl_2_ 1.25, NaHCO_3_ 25, and d-glucose 11, pH 7.4) at 37 °C for the assessment of changes in isometric tension. Following equilibration for at least 60 min at a stable resting tension of 1 g, rings were exposed to 80 mM of KCl-containing Krebs solution. Thereafter, rings were contracted with phenylephrine (1 μM) before the induction of a relaxation to acetylcholine (1 μM), to clarify the presence of a functional endothelium. After washout and a 30-min resting time, rings were contracted with phenylephrine (1 μM) prior to the construction of a concentration-relaxation curve in response to acetylcholine (1 nM–10 μM) or sodium nitroprusside (0.1 nM–10 μM). In some experiments, rings were exposed to an inhibitor for 20 min before the addition of phenylephrine. To evaluate NO-mediated relaxation, rings were incubated in the presence of indomethacin (10 μM), and TRAM-34 (1 μM) plus UCL-1648 (1 μM) to exclude the formation of vasoactive prostanoids and EDH-mediated relaxation, respectively. The EDH-mediated relaxation was evaluated in rings incubated with indomethacin and N^ω^-nitro-l-arginine (L-NA; 300 μM) to exclude the formation of vasoactive prostanoids and NO, respectively. To evaluate EDCFs, rings were exposed to L-NA and TRAM-34 plus UCL-1648 for 20 min to prevent the formation of NO and EDH, respectively before the induction of about a 20–30% pre-contraction with phenylephrine followed by the construction of a concentration-contraction curve to acetylcholine.

### Statistical analysis

Values are expressed as mean ± SEM of different rats. Statistical analysis was assessed by one-way analysis of variance followed by Tukey’s multiple comparison post hoc test using GraphPad Prism (Version 7). The differences between groups were considered statistically significant at *P* < 0.05.

## Results

### Effect of empagliflozin treatment on metabolic parameters in ZSF1 rats

Plasma parameters have indicated an increased level of glycemia, plasma proteins and albumin, AST, ALT and ALP whereas plasma creatinine and bilirubin were decreased in the obese ZSF1 group compared to the lean control group (Table 1). The empagliflozin treatment significantly decreased glycemia and ALP in the obese ZSF1 group but not in the lean control group except creatinine, which was slightly but significantly decreased (Table 1). In addition, the empagliflozin treatment increased urea in both the lean control and obese ZSF1 groups (Table 1). The obese ZSF1 group had an altered plasma lipid profile with increased levels of total cholesterol, HDL, LDL and triglycerides compared to the lean control group (Table 1). Among them, the level of total cholesterol and triglycerides was significantly decreased by the empagliflozin treatment (Table 1). Blood ketone levels were similar in the lean control and obese ZSF1 groups, and both were significantly increased by the empagliflozin treatment (Table 1). Both glycosuria and proteinuria were significantly increased in the obese ZSF1 group compared to the lean control group (Table 1). The empagliflozin treatment increased glycosuria in both the lean control and obese ZSF1 groups, and markedly reduced proteinuria in the obese ZSF1 group without affecting that in the lean control group (Table 1).

**Table 1:** Effect of a 6-week oral intake of empagliflozin on plasma, blood and urine parameters in the lean control and ZSF1 groups

	Control	Empa	ZSF1	ZSF1 + Empa
Plasma				
Glycemia (mmol/l)	11.33 ± 1.11	9.94 ± 1.30	28.07 ± 2.13*	20.81 ± 0.78*^,#^
Urea (mmol/l)	5.84 ± 0.26	8.71 ± 0.31*	6.86 ± 0.30	7.56 ± 0.28*
Plasma creatinine (µmol/l)	33.18 ± 0.46	31.23 ± 0.56*	24.21 ± 0.80*	25.37 ± 0.22
Plasma proteins (g/l)	58.89 ± 0.99	58.88 ± 0.83	64.88 ± 0.90*	64.00 ± 0.69*
Albumin (g/l)	36.49 ± 0.49	37.16 ± 0.49	39.46 ± 0.48*	39.49 ± 0.43*
Bilirubin (μmol/l)	0.57 ± 0.02	0.61 ± 0.06	0.25 ± 0.16*	0.01 ± 0.01*
AST (U/l)	130.90 ± 12.34	171.00 ± 7.80	220.60 ± 35.64*	152.20 ± 8.94
ALT (U/l)	63.78 ± 2.74	108.80 ± 7.56	161.20 ± 19.02*	131.20 ± 8.05
ALP (U/l)	62.78 ± 4.06	72.13 ± 8.60	136.60 ± 13.24*	97.11 ± 10.87^#^
Total cholesterol (mmol/l)	2.33 ± 0.06	2.38 ± 0.11	6.31 ± 0.34*	4.90 ± 0.30*^,#^
HDL cholesterol (mmol/l)	1.37 ± 0.02	1.43 ± 0.05	2.51 ± 0.09*	2.45 ± 0.08*
LDL cholesterol (mmol/l)	0.63 ± 0.03	0.69 ± 0.02	0.75 ± 0.04*	0.66 ± 0.06
Triglycerides (mmol/l)	0.48 ± 0.05	0.49 ± 0.03	6.23 ± 1.06*	2.92 ± 0.28*^,#^
Blood				
Ketone (mmol/l)	0.33 ± 0.06	0.71 ± 0.10*	0.32 ± 0.06	0.56 ± 0.10^#^
Glucose (mg/dl)	134.60 ± 9.07	134.10 ± 8.69	274.50 ± 29.41*	196.70 ± 22.16^#^
Urine				
Glycosuria (mmol)	0.89 ± 0.40	12.03 ± 1.55*	6.09 ± 0.85*	17.47 ± 0.98*^,#^
Proteinuria (g/l)	1.59 ± 0.17	0.74 ± 0.06	11.35 ± 0.84*	4.62 ± 0.44*^,#^
Volume (ml)	9.33 ± 0.94	26.22 ± 1.62*	22.11 ± 2.31*	39.40 ± 2.22*^,#^

Metabolic parameters were measured in plasma except for ketone and glucose which were determined in whole blood, and glycosuria and proteinuria which were determined in urine collected over a 24-h period. Values are shown as mean ± SEM of n = 5–10 per group

*Empa* empagliflozin, *AST* aspartate aminotransferase, *ALT* alanine aminotransferase, *ALP* alkaline phosphatase, *HDL* high density lipoprotein, *LDL* low density lipoprotein

* *P* < 0.05 vs control group and ^#^*P* < 0.05 vs ZSF1 group

### Effect of empagliflozin treatment on morphometric parameters in ZSF1 rats

The morphometric evaluation of rats and organs has indicated that the weight of several organs including spleen, liver, kidney, perirenal fat, lung, heart and body weight were higher in the obese ZSF1 group than the lean control group (Table 2). The empagliflozin treatment significantly decreased the weight of the spleen, liver, perirenal fat, lung, heart and body weight in the obese ZSF1 group (Table 2). In the control group, empagliflozin significantly decreased the weight of the heart and body (Table 2).

**Table 2:** Effect of empagliflozin on morphometric parameters of different organs and body weight in the lean control and ZSF1 groups

	Control	Empa	ZSF1	ZSF1 + Empa
Spleen (mg mm^−1^)	17.45 ± 0.38	16.55 ± 0.62	20.90 ± 0.32*	18.53 ± 0.81^#^
Liver (mg mm^−1^)	286.40 ± 12.58	264.80 ± 7.80	771.40 ± 34.64*	547.90 ± 21.37*^,#^
Kidney (mg mm^−1^)	40.52 ± 1.33	41.58 ± 1.34	55.22 ± 1.46*	54.92 ± 1.60*
Perirenal fat (mg mm^−1^)	11.32 ± 1.85	8.47 ± 1.63	183.70 ± 36.89*	100.60 ± 27.25*^,#^
Left lung (mg mm^−1^)	11.06 ± 0.20	10.65 ± 0.65	12.92 ± 0.69*	11.02 ± 0.25^#^
Dry weight of left lung (mg mm^−1^)	2.30 ± 0.03	2.16 ± 0.06	2.70 ± 0.08*	2.42 ± 0.06^#^
3 lobes of lung (mg mm^−1^)	17.94 ± 0.41	16.40 ± 0.41	21.48 ± 1.64*	17.23 ± 0.37^#^
Heart (mg mm^−1^)	31.93 ± 0.66	28.74 ± 0.75*	38.98 ± 0.95*	35.40 ± 0.63*^,#^
Tibial length (mm)	41.76 ± 0.35	42.54 ± 0.53	40.22 ± 0.42	39.89 ± 0.43
Weight (g)	407.70 ± 9.07	366.00 ± 7.67*	523.80 ± 19.93*	478.90 ± 8.73*^,#^

Organs were weighted and indexed to the respective tibial length. Values are shown as mean ± SEM of n = 7–10 per group

*Empa* empagliflozin

* *P* < 0.05 vs control group and ^#^*P* < 0.05 vs ZSF1 group

### Empagliflozin treatment reduces systolic blood pressure in obese ZSF1 rats

**Fig. 1:**
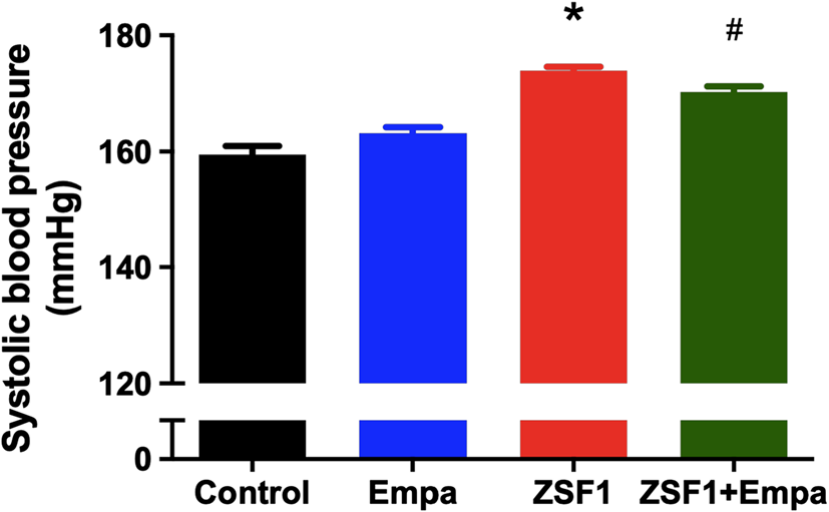
Effect of empagliflozin treatment on systolic blood pressure in the lean control and the ZSF1 groups. Values are shown as mean ± SEM of n = 9–10 per group. *P < 0.05 vs control group and ^#^*P* < 0.05 vs ZSF1 group

Systolic blood pressure was higher by about 14.5 mmHg in the obese ZSF1 group compared to that in the lean control group (Fig. 1). The empagliflozin treatment significantly reduced systolic blood pressure by about 3.6 mmHg (reduction from 173.9 ± 0.7 to 170.3 ± 0.9 mmHg) in the obese ZSF1 group after 5 weeks without affecting that in the lean control group (Fig. 1).

### Empagliflozin prevents alteration of heart structure and function in obese ZSF1 rats

**Fig. 2:**
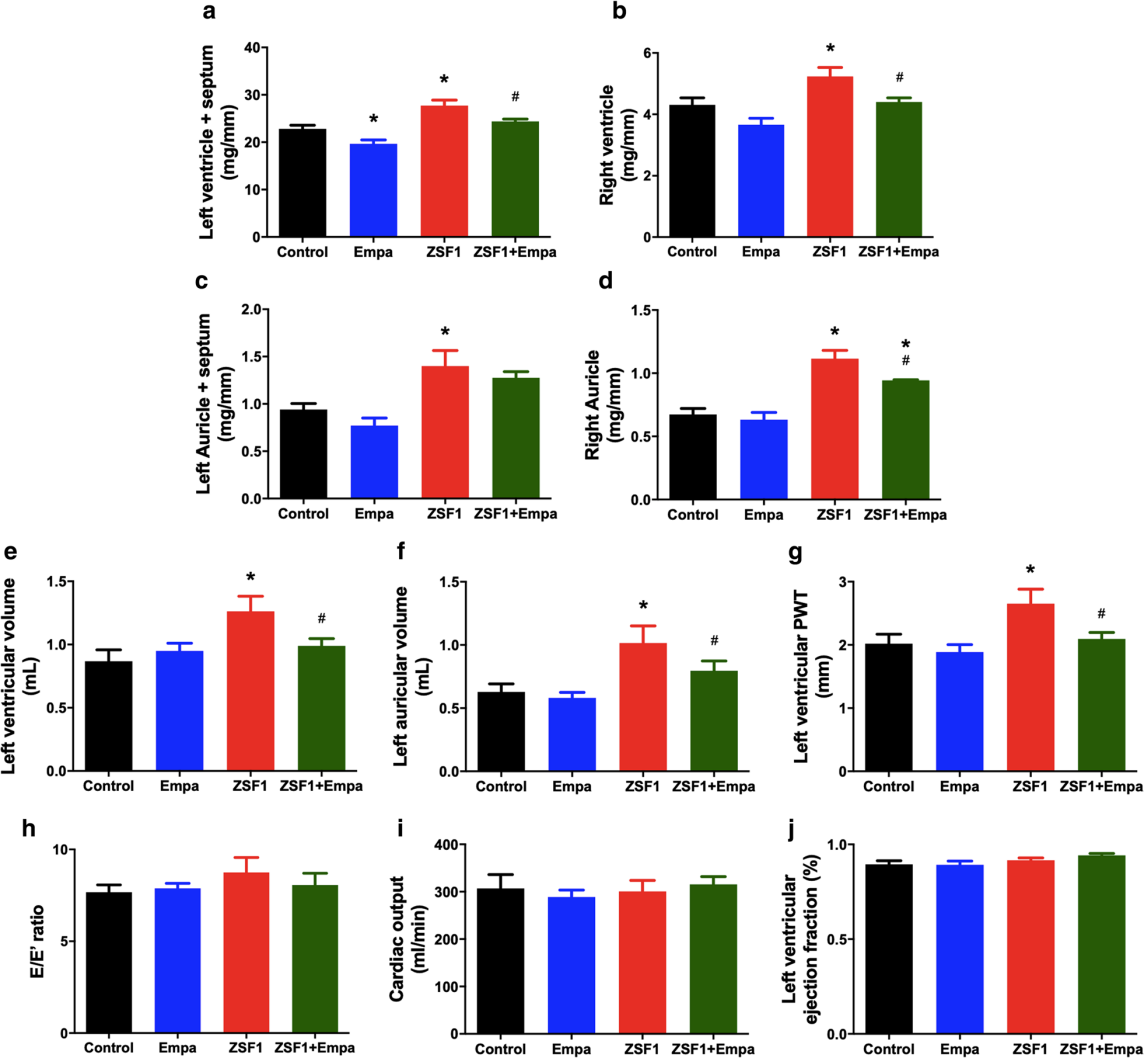
Oral intake of empagliflozin prevents heart remodeling in ZSF1 rats. **a**–**d** All the four cavities of heart (left ventricle and septum, right ventricle, left auricle and septum and right auricle) were weighted and indexed to the respective tibial length. **e**–**j** The different cardiac markers related to cardiac function and morphology were assessed by echocardiography. Values are shown as mean ± SEM of n = 6–10 per group. *P < 0.05 vs control group and ^#^*P* < 0.05 vs ZSF1 group

Evaluation of the structure of the different parts of the heart by weight and echocardiography has indicated that the left ventricle plus septum, right ventricle, left auricle plus septum and right auricle weights, the left ventricular and auricular volume and the left ventricular posterior diastolic wall thickness (PWT) were increased in the obese ZSF1 group compared to those of the lean control group (Fig. 2). The empagliflozin treatment significantly reduced the left ventricle plus septum, right ventricle and right auricle weights, and also the left ventricular and auricular volumes in the obese ZSF1 group (Fig. 2). The empagliflozin treatment also reduced the left ventricle plus septum weight in the control lean group, whereas the other parameters were not affected (Fig. 2).

The cardiac output and left ventricular ejection fraction were similar in the lean control and the obese ZSF1 groups, and not affected by the empagliflozin treatment (Fig. 2). The E/E′ ratio, an indicator of the function of left ventricle filling, was slightly increased in the obese ZSF1 group compared to that of the lean control group, however, this effect did not reach statistical significance (Fig. 2).

### Empagliflozin treatment improves endothelium-dependent relaxations and reduces endothelium-dependent contractile responses in ZSF1 rats: role of cyclooxygenases

**Fig. 3:**
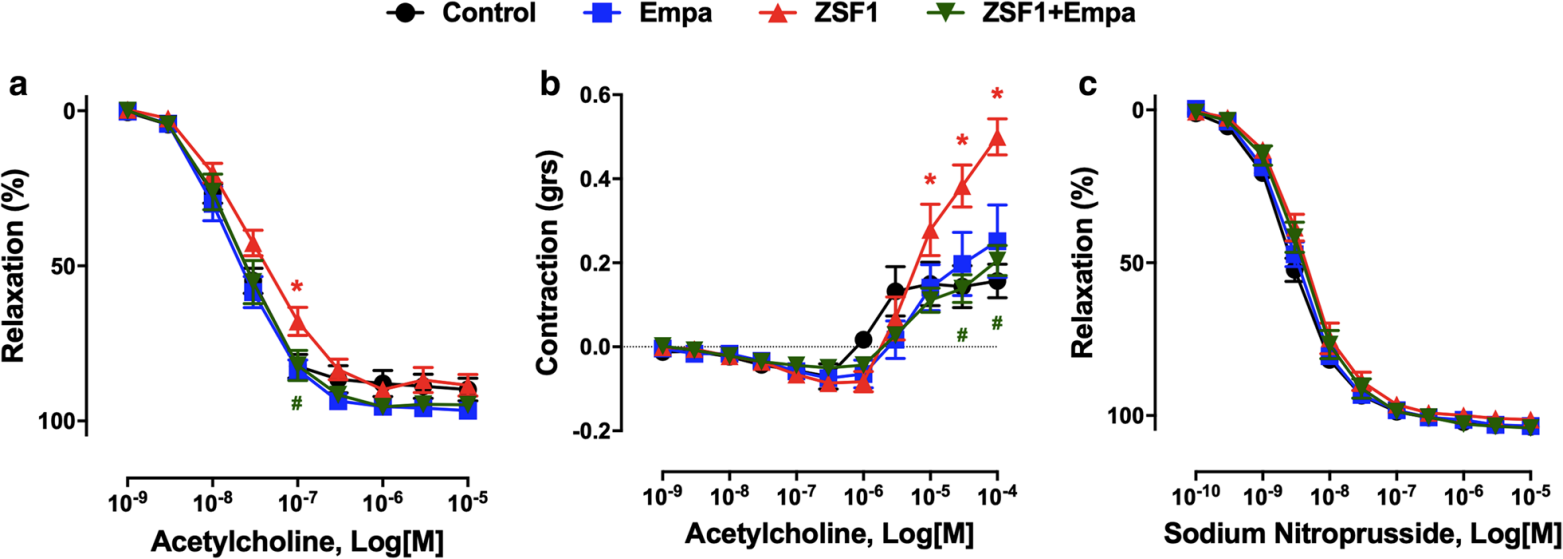
Effect of empagliflozin treatment on the endothelium-dependent relaxation and endothelium-dependent contractile response to acetylcholine in the lean control and ZSF1 groups. Arterial rings from the main mesenteric artery with endothelium were suspended in organ baths containing oxygenated Krebs buffer. For concentration-relaxation curves, rings were precontracted with phenylephrine (1 μM) before the addition of increasing concentrations of either acetylcholine (**a**) or the NO donor sodium nitroprusside (**c**). **b** Endothelium-dependent contractile responses (EDCF) were studied in the presence of N^ω^-nitro-l-arginine (300 μM) and UCL-1684 plus TRAM-34 (1 μM each) to prevent the formation of NO- and EDH-mediated relaxations, respectively. Rings were precontracted to about 20–30% of the maximal contraction with phenylephrine before the addition of increasing concentrations of acetylcholine. Values are shown as mean ± SEM of 8–10 rats per group. *P < 0.05 vs control group and ^#^*P* < 0.05 vs ZSF1 group

The endothelial and vascular function were assessed by vascular reactivity studies of the main mesenteric artery. The concentration-dependent relaxation curve to acetylcholine was slightly but significantly shifted to the right in mesenteric artery rings with endothelium of the obese ZSF1 compared to that of the lean control group (Fig. 3a). The blunted endothelium-dependent relaxation in the ZSF1 group was associated with increased endothelium-dependent contractile responses to acetylcholine (Fig. 3b). The empagliflozin treatment restored normal endothelium-dependent relaxations and blunted endothelium-dependent contractile responses to acetylcholine (Fig. 3a, b). The empagliflozin treatment did affect neither relaxations nor the small endothelium-dependent contractile responses to acetylcholine in the control lean group (Fig. 3a, b). In addition, relaxations to the NO donor sodium nitroprusside were similar in all groups (Fig. 3c).

**Fig. 4:**
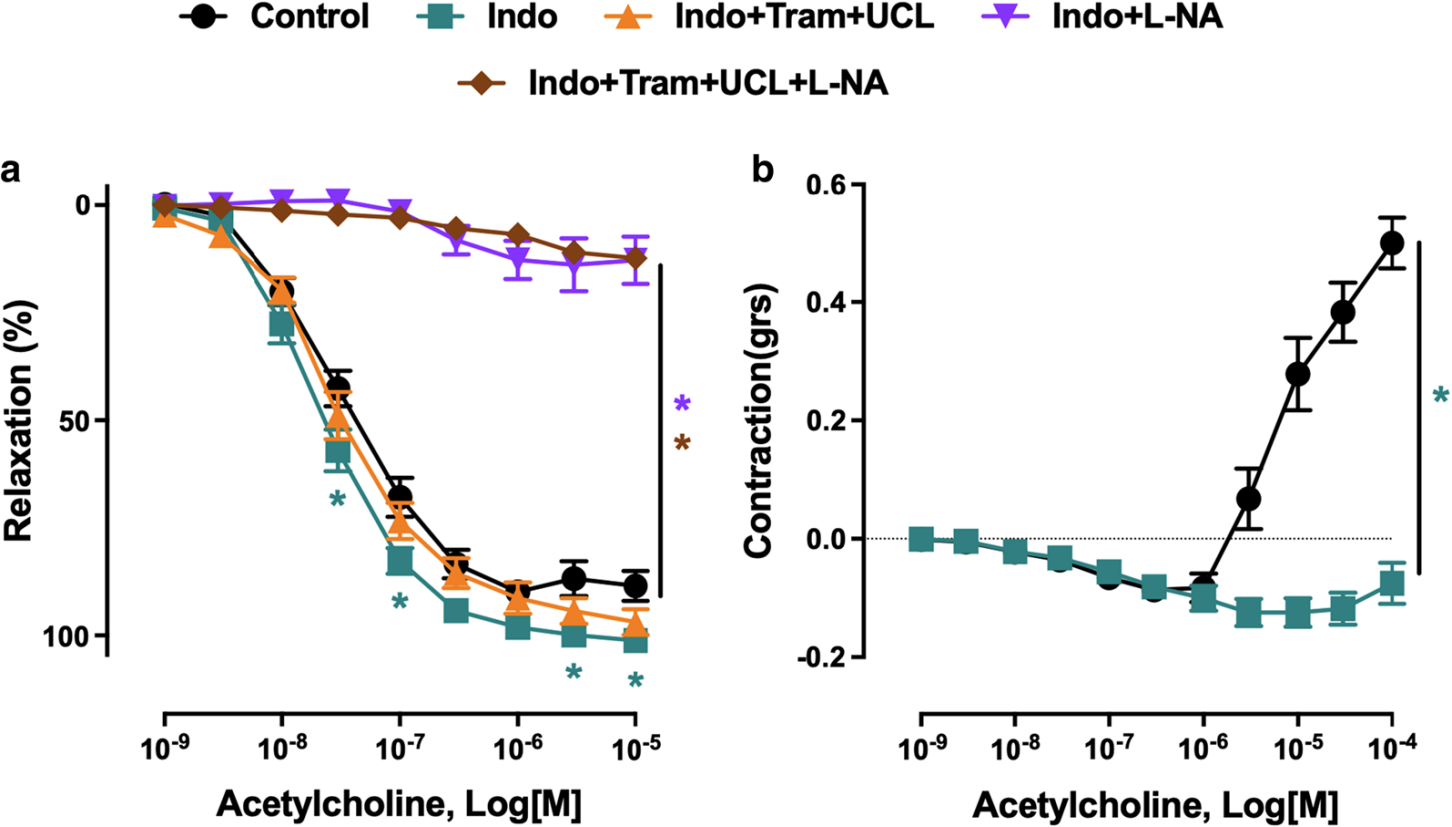
Characterization of endothelium-dependent relaxation and contractile responses to acetylcholine in mesenteric artery rings with endothelium of the ZSF1 group. Arterial rings from the main mesenteric artery with endothelium were suspended in organ baths containing oxygenated Krebs buffer. Pharmacological inhibitors were added 20 min before the contraction to phenylephrine to assess the role of the NO-mediated component (N^ω^-nitro-l-arginine, L-NA, 300 μM), the EDH-mediated component (UCL-1684 plus TRAM-34, UCL + Tram, 1 μM each), and the formation of vasoactive prostanoids (indomethacin, Indo, 10 μM). Rings were contracted with phenylephrine (1 μM) (**a**) or to about 20-30% of the maximal contraction (**b**) before the addition of increasing concentrations of acetylcholine. Values are shown as mean ± SEM of n = 8–10. *P < 0.05 vs control group

The characterization of the blunted endothelium-dependent relaxation to acetylcholine in mesenteric artery rings of the ZSF1 group has indicated a slight but significant increased relaxation in the presence of indomethacin, a non-selective inhibitor of cyclooxygenases indicating the involvement, to some extent, of vasoconstrictor prostanoids (Fig. 4a). The role of endothelial NO in the relaxation to acetylcholine was assessed using the NO synthase inhibitor N^ω^-nitro-l-arginine, and that of EDH by using TRAM-34 and UCL-1684, inhibitors of intermediate and small calcium-dependent K^+^ channels, respectively, involved in EDH. Although the addition of TRAM-34 and UCL-1684 to indomethacin affected only slightly the relaxation to acetylcholine, the response was abolished in the presence of N^ω^-nitro-l-arginine demonstrating the exclusive involvement of NO (Fig. 4a). In addition, the endothelium-dependent contractile response to acetylcholine in mesenteric artery rings of the obese ZSF1 group was abolished in the presence of indomethacin (Fig. 4b).

### Empagliflozin prevents the expression of senescence and pro-atherothrombotic markers, and also SGLT1 and 2 at arterial sites at risk

**Fig. 5:**
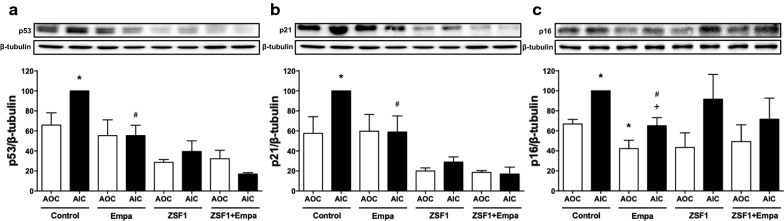
Effect of empagliflozin treatment on the expression level of senescence markers (p53, p21 and p16) in segments of the outer curvature (AOC), an arterial site at low risk, and in those of the inner curvature (AIC) of the aortic arch, an arterial site at high risk, in the lean control and ZSF1 groups as assessed by Western blot analysis. Results are shown as representative immunoblots (upper panels) and corresponding cumulative data (lower panels). Values are shown as mean ± SEM of n = 3–4 per group. *P < 0.05 vs AOC of control group and ^#^*P* < 0.05 vs AIC of control group and ^+^*P* < 0.05 vs AOC of Empa group

Since senescence has been identified as an early event promoting endothelial dysfunction [17], the expression level of the senescence markers p53, p21 and p16 was evaluated by Western blot analysis in the outer aortic arch curvature, an arterial site exposed to a high level of shear stress and at low risk, and the inner aortic arch curvature, an arterial site exposed to a low level of shear stress and at high risk [32]. The expression level of p53, p21 and p16 were significantly higher in the inner than outer aortic arch curvatures in the lean control group (Fig. 5). The empagliflozin treatment reduced the expression level of p53, p21 and p16 in the inner curvature to a similar level as those observed in the outer curvature of the aortic arch in the lean control group (Fig. 5). The empagliflozin treatment also reduced significantly the expression level of p16 in the outer aortic arch curvature whereas those of p53 and p21 were not affected in the lean control group (Fig. 5).

In contrast to the lean control group, the obese ZSF1 group showed low levels of p53 and p21 which were similar in the inner and outer aortic arch curvatures and not affected by the empagliflozin treatment (Fig. 5a, b). Regarding p16 levels, an increased level was observed in the inner compared to the outer curvatures, however, this difference did not reach statistical significance and was not affected by the empagliflozin treatment (Fig. 5c).

**Fig. 6:**
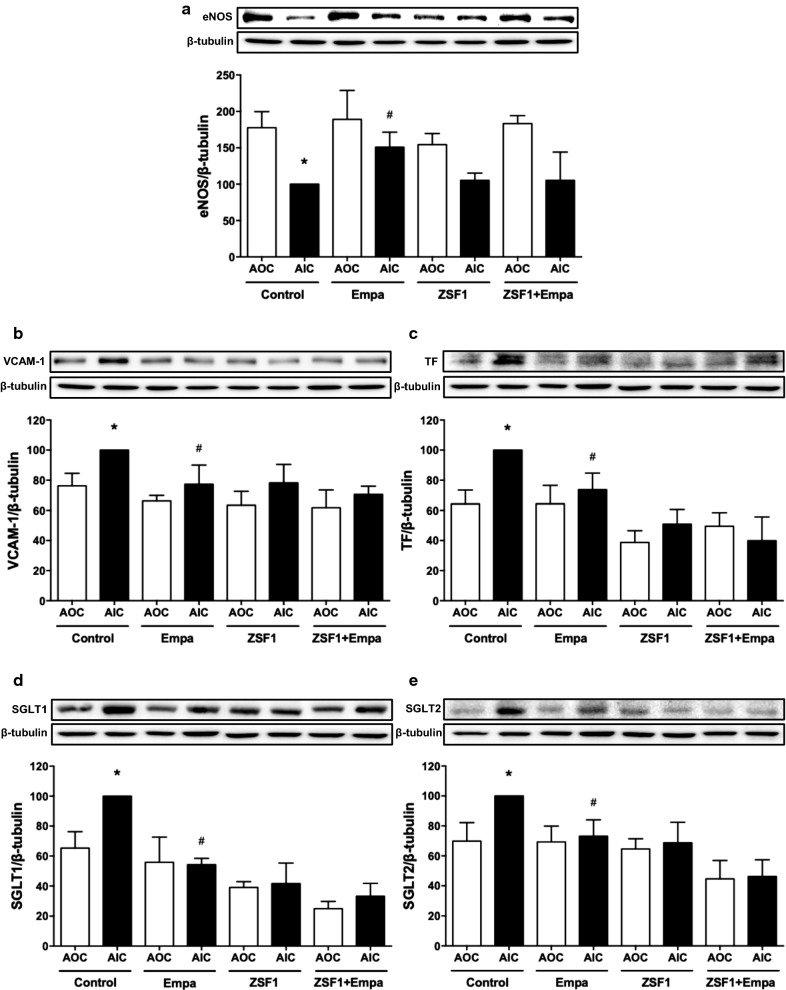
Effect of empagliflozin treatment on the expression level of eNOS (**a**), VCAM-1 (**b**), TF (**c**), SGLT1 (**d**) and SGLT2 (**e**) in segments of the AOC and in those of the AIC of the lean control and ZSF1 groups as assessed by Western blot analysis. Results are shown as representative immunoblots (upper panels) and corresponding cumulative data (lower panels). Values are shown as mean ± SEM of n = 3–4 per group. *P < 0.05 vs AOC of control group and ^#^*P* < 0.05 vs AIC of control group

To evaluate the endothelial dysfunction at arterial sites at risk, the expression level of pro-atherothrombotic markers including eNOS, VCAM-1 and tissue factor, the initiator of the coagulation cascade, was assessed in the inner and outer aortic arch curvatures by Western blot analysis. These investigations have indicated that eNOS is down-regulated and VCAM-1 and tissue factor are up-regulated in the inner versus outer aortic arch curvatures in the lean control group, and that these effects are normalized by the empagliflozin treatment (Fig. 6a–c). In the ZSF1 group, eNOS levels were decreased in the inner versus outer aortic arch curvatures, but this effect did not reach statistical significance (Fig. 6a). The levels of VCAM-1 and tissue factor were similar in the inner and outer aortic arch curvatures of the ZSF1 group, and not affected by the empagliflozin treatment (Fig. 6b, c).

Of interest, an increased expression level of SGLT1 and SGLT2 (immunoreactive bands of about 70 kDa) were observed in the inner compared to the outer aortic arch curvatures in the lean control group (Fig. 6d, e). The empagliflozin treatment normalized the expression level of SGLT1 and SGLT2 in the inner aortic arch curvature to a level similar as that observed in the outer aortic arch curvature (Fig. 6d, e). Similar expression levels of SGLT1 and 2 were observed in the inner and outer aortic arch curvatures in the ZSF1 group and the empagliflozin-treated ZSF1 group (Fig. 6d, e).

## Discussion

The major findings of the present study indicate that the selective SGLT2 inhibitor empagliflozin improves systolic blood pressure, heart remodeling and endothelial dysfunction in an experimental model of metabolic syndrome with HFpEF, the ZSF1 rat. The protective effect of empagliflozin on the heart of the ZSF1 rat with HFpEF involves normalization of the heart weight with an improvement of the left ventricle weight and volume, and also of the posterior wall thickness. The protective effect of empagliflozin on the endothelial function in the mesenteric artery involves normalization of NO-mediated endothelium-dependent relaxations and prevention of endothelium-dependent contractile responses to acetylcholine most likely by targeting the cyclooxygenase pathway. A protective effect of empagliflozin is also observed at arterial sites at risk (inner versus outer curvatures of the aortic arch) in the lean control group as indicated by the normalization of the expression level of senescence markers (p53, p21, p16), atherothrombotic markers, and SGLT1 and 2. Moreover, the empagliflozin treatment resulted in improved glycemic level, lipid profile and body weight. Altogether, the present findings indicate that the highly selective SGLT2 inhibitor is able to target several major components of the metabolic syndrome in the ZSF1 rat to improve the function of the cardiovascular system.

The EMPA-REG OUTCOME, the CANVAS and the DECLARE-TIMI58 trials showed that SGLT2 inhibitors reduced the risk of cardiovascular death or hospitalization for heart failure in T2D individuals with established CVD [18–20]. The subsequent analysis of the EMPA-REG OUTCOME data indicated that the cardiovascular benefits of empagliflozin is independent of glycemic control [21]. More recently, the DAPA-Heart Failure trial indicated that among patients with heart failure and a reduced ejection fraction, dapagliflozin reduced the risk of worsening heart failure or death from cardiovascular causes regardless of the presence or absence of diabetes [33]. Altogether, these findings suggest that SGLT2 inhibitors act on the cardiovascular system possibly through glucose-independent mechanisms, which however remain to be elucidated. Several potential mechanism, besides glycemic control, have been suggested such as the involvement of visceral adiposity, body weight, hyperinsulinemia, blood pressure, arterial stiffness, lipid profile, and albuminuria [34]. In addition, SGLT1 and 2 expression has been observed in cultured and native ECs under pathological conditions such as hyperglycemic state and oxidative stress, promoting endothelial senescence and dysfunction subsequent to excessive glucose entry [30]. Since all of these effects were inhibited by empagliflozin, SGLT2 inhibitors may possibly also contribute to protect the cardiovascular system by targeting the pivotal endothelial function [30]. Therefore, the present study has evaluated the impact of empagliflozin on the cardiovascular system in an experimental model of metabolic syndrome with HFpEF, the ZSF1 rat, which combines several major cardiovascular risk factors including obesity, hypertension, diabetes and dyslipidemia [35–37].

After 18 weeks, the obese ZSF1 rat showed many characteristics of metabolic risk such as visceral obesity as indicated by increased perirenal fat, elevated body weight, hyperglycemia, and dyslipidemia characterized by high levels of total cholesterol, LDL and triglycerides. In addition, an increased level of systolic blood pressure was observed in the obese ZSF1 rat compared to the lean rat. The present findings indicate a beneficial effect of the empagliflozin treatment promoting a reduction of the body weight and the weight of perirenal fat, and hyperglycemia, and an improvement of the lipid profile as well as of systolic blood pressure in the obese ZSF1 rat. Previous studies have also shown that empagliflozin reduced body weight and fat associated with an increased urinary glucose excretion in an animal model of diet-induced obesity [38], lowered plasma cholesterol and liver triglyceride levels in pre-diabetic ob/ob^−/−^ mice [39], and decreased blood pressure by about 15 mmHg in the Cohen-Rosenthal diabetic hypertensive rat [25].

Consistent with previous studies, the obese ZSF1 rat, an experimental model of HFpEF, is characterized by pronounced heart remodeling affecting the left and right heart with a preserved heart function as indicated by normal cardiac output and ejection fraction. The present findings indicate that the empagliflozin treatment was able to prevent the hypertrophy of both the left and right heart as indicated by the normalization of the weight of the left and right ventricles and the right auricle, and also of the heart remodeling as indicated by the normalization of the volume of the left ventricle and auricle, and the posterior wall thickness of the left ventricle. A beneficial effect of empagliflozin on the heart has also been observed previously since empagliflozin reduced left ventricle weight, cardiomyocyte size, cardiac interstitial fibrosis and macrophage infiltration in genetic pre-diabetic rats with metabolic syndrome [24], improved cardiac function and ATP production in db/db mice associated with preserved cardiac glucose and lipid metabolism [40] and improved diastolic function, mitochondrial expansion, and myocardial fibrosis in female db/db mice [41]. In addition, Connelly et al. observed that empagliflozin preserved lung weight, ameliorated diastolic dysfunction, and attenuated cardiac hypertrophy using a rat model of uninephrectomy treated with deoxycorticosterone acetate and 1% NaCl water to induce HFpEF [42].

Chronic inhibition of SGLT2 decreases extracellular fluid and plasma volumes as was obvious in the EMPA-REG OUTCOME trial with a 5.2% rise in hematocrit level at the end of the study [18]. The diuretic effect subsequent to inhibition of the SGLT2-mediated glucose uptake in the kidney proximal tube will further reduce central aortic pressure and afterload, resulting in improved left ventricular function, and decreased cardiac workload and myocardial oxygen demand [43, 44]. Preload reduction by lower plasma volume most likely acts synergistically with afterload reduction to promote a reduction of cardiac events, especially in individuals with diabetes and impaired left ventricular function, ischemic heart disease, or congestive HF [43, 44].

Endothelial dysfunction caused by hyperglycemia is a critical initiator of the development of macro- and micro-vascular complications in T2D with high metabolic risk [7, 45]. Previous studies have indicated that hyperglycemia-related endothelial dysfunction is characterized often by blunted NO and EDH components of the endothelium-dependent relaxation as observed in the rat arterioles [14], rat aorta [13], and rabbit aorta [12]. In the obese ZSF1 rat, the endothelial dysfunction of the main mesenteric artery is characterized by blunted endothelium-dependent relaxations and the appearance of endothelium-dependent contractile responses to acetylcholine. Moreover, the characterization of the endothelial dysfunction to acetylcholine indicated that the NO component is significantly reduced in the mesenteric artery of the obese ZSF1 rat. Interestingly, empagliflozin treatment restored the protective endothelial function in the mesenteric artery as indicated by normal endothelium-dependent relaxations and blunted endothelium-dependent contractile responses to acetylcholine most likely by preventing the formation of cyclooxygenase-derived contractile prostanoids. Experimental investigations have also shown that empagliflozin improved endothelium-dependent relaxations in streptozotocin‐induced diabetic rats and Zucker diabetic fatty rats [22, 27]. Canagliflozin has also been shown to increase SNP-dependent relaxation in coronary arteries from diabetic mice subsequent to vascular smooth muscle hyperpolarization involving potassium channels [46] and Ipragliflozin improved endothelium-dependent vasodilation in streptozocin-induced diabetic mice, in part, by an improvement of eNOS function as suggested by an increased phosphorylation level of eNOS at the activator site Ser1177 and Akt [47].

Premature endothelial senescence as indicated by senescence-associated β-galactosidase (SA-β-gal), p53 and p16 staining was observed in the aortas obtained from Zucker diabetic rats [48]. SA-β-gal-positive staining was also observed in atherosclerotic lesions of the human thoracic aorta at the intimal side as well as at sites of disturbed blood flow including branches and bifurcations [49, 50]. Since the selective p53 overexpression in ECs resulted in impaired endothelium-dependent relaxation and NO bioavailability [17], premature endothelial senescence most likely acts as an upstream signaling event to promote endothelial dysfunction. Although the lean control ZSF1 rat is characterized by normal heart structure and function, and endothelial function in the mesenteric artery, a more precise analysis of the vascular tree has provided evidence of premature senescence at arterial sites at risk. Indeed, an increased level of senescence markers p53, p21 and p16 was observed in the inner compared to the outer aortic arch curvature, a potential site of early atherogenesis characterized by disturbed flow and low shear stress [32]. Moreover, the premature senescence was associated with the appearance of pro-atherothrombotic signals as indicated by a decreased expression level of eNOS and increased levels of tissue factor, the main activator of the coagulation cascade, and VCAM-1 involved in the recruitment of monocytes into the arterial wall. Furthermore, an increased expression level of SGLT1 and SGLT2 was observed at the arterial site at risk supporting the concept that possibly an excessive entry of glucose via SGLTs might contribute to promote structural and functional changes favoring the pathologic development of the arterial site at risk. The present findings indicate that the empagliflozin treatment was able to normalize the expression level of senescence markers, eNOS, tissue factor, VCAM-1 and SGLT1 and 2 in the inner aortic arch curvature to levels similar to those in the outer aortic arch curvature, which is protected by the exposure to high levels of shear stress. Thus, these observations suggest that empagliflozin might possibly contribute to protect arterial sites of atherogenesis by blunting the SGLT2-mediated pro-senescent and pro-atherothrombotic responses [30]. The fact that no such differences in the expression level of pro-atherothrombotic markers are observed in the inner and outer curvature of the obese ZSF1 rat suggests that the responsiveness of the arterial wall to local blood flow behavior appears to be altered possibly due to the chronic exposure of metabolic stress.

## Conclusions

The study indicates that empagliflozin prevents hypertrophy and remodeling of the heart, and also endothelial dysfunction as indicated by an improved NO-mediated relaxation and the prevention of cyclooxygenases-mediated endothelium-dependent contractile responses in an experimental model of metabolic syndrome with HFpEF. The beneficial effect involves an improvement of systolic blood pressure, glycemia, the lipid profile and body weight, and also of premature vascular senescence at arterial sites at risk.

## Data Availability

Not applicable.

## Abbreviations

AIC: Inner curvature of the aortic arch
ALP: Alkaline phosphatase
ALT: Alanine aminotransferase
AOC: Outer curvature of the aortic arch
AST: Aspartate aminotransferase
CVD: Cardiovascular disease
ECs: Endothelial cells
EDCFs: Endothelium-dependent contractile responses
EDH: Endothelium-dependent hyperpolarization
empa: Empagliflozin
eNOS: Endothelial nitric oxide synthase
HDL: High density lipoprotein
HF: Heart failure
HFpEF: Heart failure with preserved ejection fraction
L-NA: N^ω^-nitro-l-arginine
LDL: Low density lipoprotein
NO: Nitric oxide
PWT: Posterior diastolic wall thickness
SA-β-gal: Senescence-associated β-galactosidase
SGLT: Selective sodium–glucose cotransporter
T2D: Type 2 diabetes
TBS: Tris-buffered saline
TF: Tissue factor
ZSF1: Obese Zucker diabetic fatty/spontaneously hypertensive heart failure F1 hybrid
